# Association between active/passive Involution and lying flat among college students: the mediation role of perceived stress and anxiety

**DOI:** 10.1186/s12889-025-21994-z

**Published:** 2025-06-02

**Authors:** Lei Yang, Yunbing Zhang, Dongjun Zhang, Lujun Shen, Hui Feng

**Affiliations:** 1 School of Psychology, Xinxiang Medical University, Xinxiang City, Henan Province China; 2 Faculty of Psychology, Tianjin Normal University, Tianjin, 300387 China; 3 School of Education, Hebei University, Baoding City, Hebei Province China; 4 The Third Affiliated Hospital of Xinxiang Medical University, Xinxiang City, Henan Province China

**Keywords:** Active Involution, Passive Involution, Perceived stress, Anxiety, Lying flat

## Abstract

**Background:**

In recent years, involution and lying flat have become popular in China. Involution is defined as the phenomenon in which people are actively or passively involved in irrational competition for limited social resources. Lying flat refers to the state in which people choose to give up their efforts and passively escape in the face of social pressure and continuous competition. Should involution lead to lying flat in college students? This study aimed to provide empirical support for examining the potential mechanism between active/passive involution and lying flat.

**Methods:**

A cross-sectional survey of 1003 college students was conducted in Henan Province, China. Participants completed the Involution Behavior Scale, Perceived Stress Scale, Generalized Anxiety Disorder-7 Scale and Lying Flat Tendency Scale through Sojump platform. Correlation and mediation models were tested using SPSS 24.0 and PROCESS macro.

**Results:**

The association between passive involution and lying flat was not only mediated through perceived stress and anxiety separately but also sequentially mediated through perceived stress and anxiety (Passive involution→ Perceived stress→ Lying flat: effect size = 0.075, 95% CI [0.042,0.113]; Passive involution→ Anxiety→ lying flat: effect size = 0.014, 95% CI [0.000, 0.031]; Passive involution→ Perceived stress→ Anxiety→ lying flat: effect size = 0.019, 95% CI [0.000, 0.039]). Active involution not only directly predicts lying flat but also indirectly predicts it through perceived stress (direct effect size = -0.301, 95% CI [-0.359, -0.242]; indirect effect size = -0.035, 95% CI [-0.055, -0.017]).

**Conclusions:**

The results reveal the influence of active/passive involution on college students’ lying flat and the mediating role of perceived stress and anxiety. The findings can provide new insights into the relationship between involution and lying flat, as well as helping students better adapt to academic learning.

## Introduction

### Involution

Over the past 40 years, China has experienced rapid economic development and social transformation. China’s social changes, such as cultural values and motivations, have had a profound impact on culture [1]. As an important product of changing times, involution has attracted the attention of netizens and researchers in recent years. Since 2020, the word “involution” has been widely discussed on the internet and was selected as one of the “Top 10 Chinese internet buzzwords” [2]. The “996” work system in the internet industry (starting work at 9 AM and ending work at 9 PM, working six days a week) reflects the prevalent culture of overtime work and, to a certain extent, contributes to the popularity of the word “involution”.

Involution is defined as the phenomenon in which people are actively or passively involved in irrational competition for limited social resources [3]. Involution implies irrational and inefficient competition and becomes a “trap” for people to compare their efforts with each other in an environment full of uncertainty. The perception of group coinvolution gradually evolves into a social atmosphere, which causes people to adopt coinvolutionary behaviors [4].

The massification of higher education has been consistent with the intensification of positional competition among graduates [5, 6]. Since 1999, the Chinese government has begun to expand the scale of enrollment in higher education. The fierce competition for employment forces college students to become involutionary. Education becomes one of the areas where involution is likely to occur, and students are highly sensitive to involution [7]. A study involving 402 college students using mediation analysis found the COVID-19 pandemic has increased the likelihood of competition for employment, leading to more intense involution among students [8]. In order to obtain high-quality education resources, the phenomenon of involution is increasingly showing a trend toward younger age groups, extending even to children [9].

Involvement may have impacts on society and individuals. Modelling the social dilemma of involution, a study found that from a group perspective, individual competition for social resources is meaningless in the absence of incremental resources [10]. The increasing efforts of students do not increase the total amount of social resources but increase the financial burden, academic burden and psychological pressure on students and their families. Irrational competition during the process of internal competition can also undermine trust and cooperation among group members, leading to deteriorating relationships between individuals and peers [11, 12]. Another study involving 1542 college students revealed that college students’ involution threatens social and emotional adjustment [13].

However, the concept of involution in previous studies mostly referred to passive involution. It should be noted that facing limited resources, college students are not always swayed by the trend of involution and may actively engage in involution [14]. Some studies have suggested that involution should be divided into two categories: passive involution and active involution. Passive involution means that students do not want to make an effort but are forced to participate in involution to avoid falling behind their peers. Active involution means that students actively participate in involution to gain achievements and rewards and improve their competitiveness [3, 15].

Previous studies on involution have focused mainly on theoretical rationales, analysing the effects of passive involution [16]. It is worthwhile to explore whether and how different types of involution influence “lying flat”.

### Lying flat

In April 2021, a blog post titled “Lying Flat is Justice” suddenly attracted widespread attention from the public [17]. Talk of “lying flat” has spread rapidly through China as young people contend with intense competition for jobs and performance. That year, the word “lying flat” was selected as one of the “top ten internet buzzwords” [2, 18]. According to an online survey of 240,000 people, more than 60% of subjects identified lying flat as their ideal lifestyle [18].

“Lying flat” refers to the state in which people choose to give up their efforts and passively escape in the face of social pressure and continuous competition [19]. The core characteristics of “lying flat” were determined as follows: lack of clear life goals, absence of desire for improvement, lack of motivation to make progress, reluctance to strive, and a tendency to avoid problems [19]. Those who lie flat are dominated by the motivation to avoid failure. They suppress their needs and expectations to avoid failure and show negative characteristics such as low value pursuit and achievement motivation [20]. A study involving 607 young Chinese using a questionnaire survey found that individuals with a greater tendency to lie flat experienced more negative emotions, lower well-being and fewer positive emotions [19]. On a social level, lying flat may lead to fewer available quality labourers, lower productivity and consumption levels, and a shrunk industrial economy [21].

Many studies have proposed that involution leads to lying flat [21]. For example, Ye argues that lying flat is a self-adaptation to involution. The three areas of Chinese society most prone to involution (education, housing and workplace) are also where lying flat is most prevalent [22]. Lying flat has become a negative resistance behavior of young Chinese people to the inefficient competition of involution [23], reflecting their general frustration and exhaustion. This phenomenon has attracted widespread attention from society.

Resource conservation theory has been successfully applied to everyday stress. According to resource conservation theory, when faced with a situation of sustained resource loss, resource-depleted individuals often choose a strategy of noninvestment to conserve their resource reserves [24]. Involvement means not only over effort but also inefficient or ineffective effort [25]. Involutionists have to spend many resources, such as time, energy and money, but may find it difficult to gain new resources. Individuals experiencing passive involution often feel compelled to keep up with their peers, yet they are less willing to exert effort and more likely to perceive resource loss. When faced with resource loss, they are more inclined to lie flat to protect their resources. In contrast, those with active involution are driven by personal growth and achievement, focusing on the beneficial outcomes of overcoming difficulties and demonstrating a greater willingness to invest effort. Although a large amount of resources is consumed, they may feel rewarded by even minor progress. Based on this, we propose Hypothesis 1: Passive involution positively predicts lying flat (H1a), while active involution negatively predicts lying flat (H1b).

Those with active involution are concerned with their own growth and achievement and have a greater willingness to make efforts. Although a large amount of resources are consumed, even slight progress can make them feel that they have gained enough rewards. The incidence of lying flat is likely to be lower. We proposed hypothesis 1: Passive involution positively predicts lying flat (H1a), while active involution negatively predicts lying flat (H1b).

It is important to note that the mediating mechanism between active/passive involution and lying flat remains unclear. Previous studies have suggested that perceived stress and anxiety might play a mediating role.

### Perceived stress as a mediator

Perceived stress highlights how people feel about stress, not just about stress itself [26]. It is an individual’s cognitive evaluation of situations and events beyond his or her self-ability [27]. Perceived stress may be a mediator between involution and lying flat.

First, active/passive involution may influence an individual’s perceived stress. To compete for limited resources, an increasing number of people fall into involution, leading to fierce and irrational competition. This competition can generate two types of stress: challenge stress and hindrance stress. Challenge stress is associated with opportunities for growth and development, while hindrance stress is related to obstacles and constraints that impede progress [28]. Passive involution is more likely to involve hindrance stress. A study involving 535 students through interviews and questionnaires found that students with passive involution begin to be involved in irrational competition only after seeing peers’ efforts; they start later and have a lower willingness to participate [3]. They have to constantly compare themselves with peers, which tend to be upwards comparisons. Social comparison theory suggests that upwards social comparison can lead to a sense of threat and pressure [29]. Data from the China Education Panel Study indicate that, especially among peers, there is a “comparative pressure” effect—the better their peers perform, the greater the pressure they feel [30]. Thus, passive involution may positively predict perceived stress. However, active involution is more likely to involve challenge stress, which can promote motivation and creativity [31]. Students with active involution pay more attention to achievements and rewards than social comparison [3]. Involution also has the potential to facilitate academic adjustment [13]. Active involutionists have a first-mover advantage. Even if there is peer comparison, it is more of a downwards comparison, which can lead to lower perceived stress [29]. Thus, active involution may negatively predict perceived stress.

Second, perceived stress can affect lying flat. Lying flat refers to the compromising behavior of young people when stress exceeds their personal psychological threshold. In the face of great and long-term social pressure, some young people are constantly frustrated. Individuals begin to relieve their perceived stress by giving up effort and lowering their expectations [17]. These individuals pay more attention to their own feelings and consciously move away from fierce competition [23, 32]. In summary, we proposed hypothesis 2: perceived stress plays a positive mediating role between passive involution and lying flat (H2a) but a negative mediating role between active involution and lying flat (H2b).

### Anxiety as a mediator

In addition to perceived stress, anxiety may also be a mediator between passive involution and lying flat. Anxiety is a comprehensive emotional state of tension, worry, and fear, accompanied by a series of symptoms [3]. Two studies using discourse analysis as a qualitative research method found that anxiety is the most frequently used emotional vocabulary in discourse related to involution on the internet, which may be primarily related to passive involution [33, 34]. Yi et al. reported that college students with high passive involution experience high anxiety [3]. These students who are dominated by strong external motivation worry about being surpassed by peers. They are often in a conflict between not wanting to involute and having to do so, which can easily lead to mental problems such as anxiety [35]. Furthermore, resource conservation theory suggests that emotional distress is closely related to resource loss [24]. Passive involutionists who follow others to involute lack long-term goals and tend to be disadvantaged. Their goals are mainly to avoid being surpassed by peers and not to focus on achievements and rewards. It is difficult to compensate for the resources consumed in involution. Thus, it is reasonable to assume that passive involution may predict anxiety.

Only one study explored the relationship between active involution and anxiety. Active involution includes achievement-motivated involution and reward-oriented involution. There is no significant correlation between achievement-motivated involution and anxiety, and reward-oriented involution cannot predict anxiety [3]. Active involutionists have clear goals and engage in competitive behavior earlier, making it easier to obtain new resources such as achievements and rewards. These new resources can to some extent compensate for those consumed in involution. However, due to limited resources, they are simply more likely to be rewarded but are not always satisfactorily rewarded. According to resource conservation theory, we assumed that active involution would not be significantly correlated with anxiety [24].

Second, the accumulation of anxiety over time can influence lying flat. Several young people mentioned in their narratives that long-term anxiety can lead to lying flat [21]. A study involving 88 college students revealed that individuals with high test anxiety tend to be dominated by the motivation to avoid failure rather than by the motivation to pursue success [36]. As a result, highly anxious students tend to exhibit avoidance behaviors [37] and have higher college dropout rates [38]. Research on 433 employees’ work behavior also supports this view. After anxiety accumulation to a certain extent, individuals choose to withdraw from work to relieve their anxiety [39].

Based on the literature review, it is reasonable to propose hypothesis 3: anxiety may only mediate the relation between passive involution and lying flat, and anxiety was not associated with active involution (H3).

### Serial mediation effect of perceived stress and anxiety

Su theoretically explained the logical association between involution and lying flat, which is sequentially linked through perceived stress and anxiety [21]. Previous research has indicated that involution is positively associated with anxiety. Work stress has the potential to affect physical and mental health. A study involving 179 nurses using mediation analysis found that nurses with greater perceived stress usually experience greater anxiety [40]. Another study involving 191 Iranian and 197 American undergraduate students have shown that perceived stress has a positive influence on anxiety [27, 41]. In particular, peer pressure, one of the main stressors for people, is associated with high anxiety [42]. Considering the relationship between passive involution and perceived stress as well as the effect of anxiety on students avoiding lying flat, we proposed hypothesis 4: the relationship between passive involution and lying flat is mediated sequentially through perceived stress and anxiety (H4).

## Methods

### Participants

To ensure adequate statistical power for path analysis, we adhered to the recommended ratio of 5–10 observations per parameter [43]. Given that our path analysis involved 53 parameters, we initially calculated a required sample size by multiplying the number of parameters by 10. Additionally, to enhance precision by 15% and account for potential sample attrition, we further expanded the sample size to at least 624 participants.

Participants were randomly selected from 2 colleges in Henan Province. A total of 1098 students completed four scales online through Sojump platform (https://www.sojump.com), which is a professional questionnaire website similar to Qualtrics. They can click submit only after they completing all items. Participants were excluded for selecting the same option repeatedly. 1003 valid questionnaires were retained, giving an effective response rate of 91.3%. Their age was 18.99 ± 1.08 years, 424 (42.3%) were male, and 579 (57.7%) were female.

This study was conducted in accordance with the Declaration of Helsinki and was approved by the Ethics Committee of Xinxiang Medical University. All participants gave their written informed consent and anonymously completed all questionnaires. Each participant received a reward of 2 yuan.

### Measures

#### Involution behavior scale (IBS)

This scale was developed by Yi et al. [3]. The IBS includes 13 daily behavior scenarios that were presented in both positive and passive expression. For example, I learn skills that I’m not interested in because (scenario 5). Positive expression, skills would enhance my employment competitiveness. Passive expression, others are learning these skills and I’m worried about falling behind. A study involving 669 college students categorized involution into passive involution and active involution (including achievement-motivated involution and reward-oriented involution) [44]. The response options ranged from 1 = “disagree strongly” to 5 = “agree strongly”, with higher scores indicating greater involution behavior. In the present study, Cronbach’s α was 0.904, and Confirmatory Factor Analysis demonstrated that χ²/df = 5.724, CFI = 0.908, TLI = 0.895, RMSEA = 0.069.

#### Perceived stress scale (PSS)

Perceived stress was measured using the Chinese version of PSS, which was developed by Cohen et al. and translated into Chinese by Yang and Huang [26, 45]. The PSS was designed to measure the degree to which situations in one’s life are appraised as stressful.

Participants responded to 14 items on a 5-point Likert scale (0 to 4, never to very often). A higher score on the PSS indicates greater perceived stress in daily life. In our study, Cronbach’s α was 0.835.

#### Generalized anxiety disorder-7 scale (GAD-7)

Anxiety was measured using the Chinese version of GAD-7. This scale was developed by Spitzer et al. and translated into Chinese by He et al. [46, 47]. The GAD-7 contains 7 items on a 4-point Likert scale (0 to 3, not at all to nearly every day). A higher score represents a greater level of anxiety. Cronbach’s α is 0.93. In our study, Cronbach’s *α* was 0.871.

### Lying flat tendency scale (LFTS)

The LFTS is a 6-item self-report questionnaire developed by Lu et al.[58] []. This scale was used to evaluate the tendency of young people to lie flat (e.g. Q1: I do not have any goals and pursuits for life and study. Q2: I feel that it is hard to change anything with my personal efforts, so I choose to give up the struggle). The items were rated on a 4-point Likert scale, ranging from 1 (very inconsistent) to 4 (very consistent). A higher score indicates a higher tendency for lying flat. It demonstrated good internal consistency and predictive validity. In the present study, Cronbach’s *α* was 0.721.

### Statistical methods

Statistical analyses were conducted using SPSS version 24.0. Descriptive statistics were generated to summarize the data. Additionally, Pearson correlation analyses were conducted to assess the relationships between the study variables. To assess the mediation model, we utilized the PROCESS macro for SPSS (Model 4 and 6). The 95% confidence interval (CI) was estimated using 5000 bootstrap re-samples. Effects were deemed statistically significant if the 95% CI did not include zero. Considering the distinct expressions of active and passive involution in the IBS, the existing literature that has categorized active and passive involution from both theoretical and empirical perspectives [15, 44], and the weak correlation between active involution and passive involution observed in our study, we treated active involution and passive involution as separate independent variables for the mediation model analysis. Based on the correlation analysis results, we first conducted a mediation analysis with active involution as the independent variable, lying flat as the dependent variable, perceived stress as the mediator, and gender and age as covariates (using Model 4 of PROCESS). Subsequently, we performed a serial mediation analysis with passive involution as the independent variable, lying flat as the dependent variable, perceived stress and anxiety as the mediators, and controlled for gender and age as covariates (using Model 6 of PROCESS).

## Results

### Correlation analysis

The correlation analysis results are presented in Table 1. There was a significant positive correlation between active involution and passive involution. Passive involution, perceived stress, anxiety and lying flat were significantly positively correlated with each other. Active involution was significantly and negatively correlated with perceived stress and lying flat.

**Table 1:** Correlation coefficients of key variables using pearson correlation

	M ± SD	1	2	3	4	5	6	7
1gender	1.58 ± 0.49	1						
2age	18.99 ± 1.08	-0.038*	1					
3 Active involution	4.19 ± 0.60	0.273**	-0.026	1				
4 Passive involution	3.12 ± 0.97	0.052	-0.002	0.133^**^	1			
5 Perceived stress	2.88 ± 0.48	0.034	-0.032	-0.131^**^	0.361^**^	1		
6 Anxiety	1.84 ± 0.53	0.001	-0.004	-0.026	0.363^**^	0.637^**^	1	
7 Lying flat	2.09 ± 0.59	-0.104**	0.074*	-0.344**	0.127**	0.268**	0.231**	1

Note: * *P* < 0.05, ** *P* < 0.01, ***P < 0.001, the same below

**Fig. 1:**
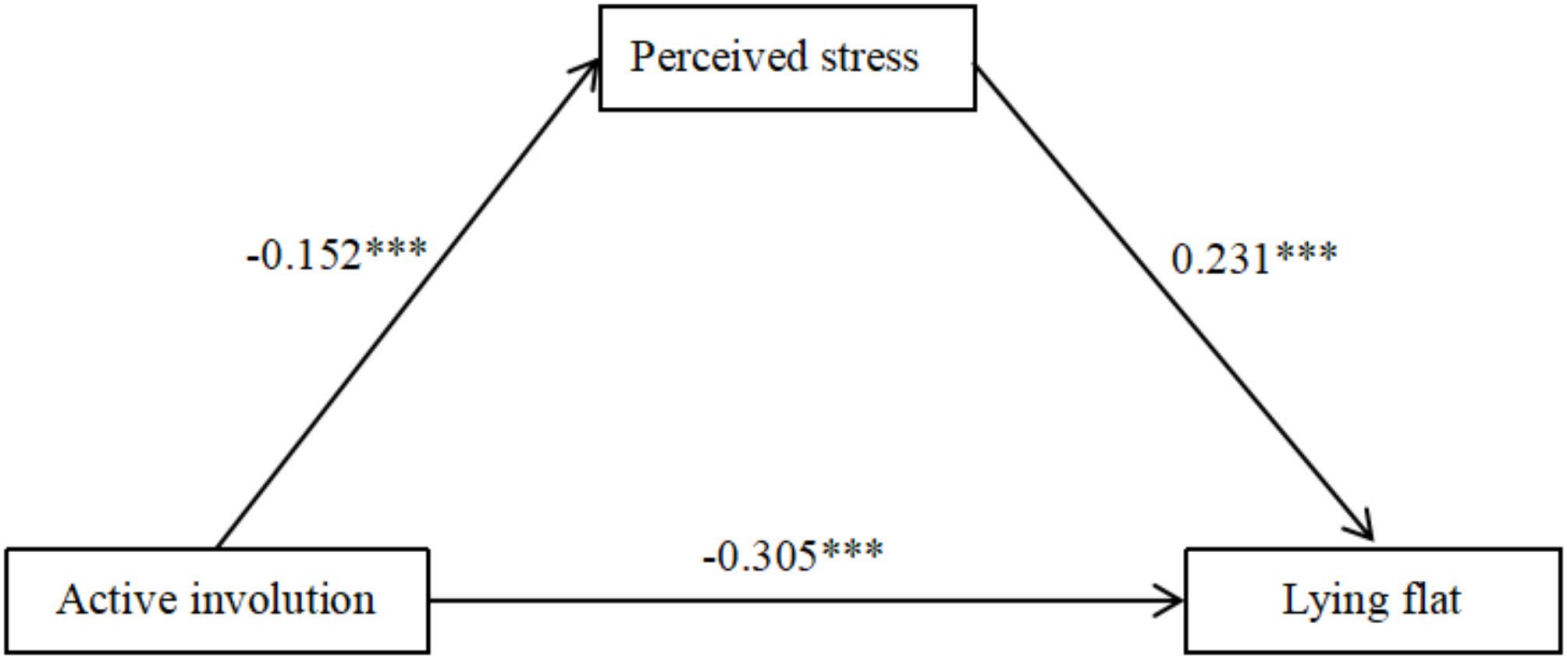
Mediation of perceived stress between active involution and lying flat

According to the significance of correlations among active involution, perceived stress and lying flat, Model 4 of the PROCESS macro was adopted to test the mediated model. We included active involution as the independent variable, lying flat as the dependent variable, perceived stress as the mediator, and controlled for gender and age as covariates. As Table 2 illustrates, active involution was negatively associated with lying flat (*β* = −0.305, *P* < 0.001), and H1b was supported. The results also showed that active involution negatively predicted perceived stress (*β* = -0.152, *P* < 0.001), and perceived stress positively predicted lying flat (*β* = 0.231, *P* < 0.001). For the indirect effect, the 95% bootstrap confidence interval (effect size = -0.035, 95% CI [-0.055, -0.017]) without “zero” indicates a significant mediation effect, and H2b was supported. The ratio of indirect effects to total effects was 10.3%. The direct effect of active involution on lying flat was also significant (effect size = -0.301, 95% CI [-0.359,-0.242]). Figure 1 displays the mediated model.

**Table 2:** Regression analysis of variable relationships in mediation model

Outcome variable	Predictor variables	*R*	*R*²	F	β	t
Perceived stress		0.153	0.023	7.974***		
	Age				-0.033	-1.067
	Gender				0.074	2.279*
	Active involution				-0.152	-4.665***
Lying flat		0.418	0.175	52.906***		
	Age				0.072	2.506*
	Gender				-0.026	-0.874
	Active involution				-0.305	-10.099***
	Perceived stress				0.231	7.941***

**Fig. 2:**
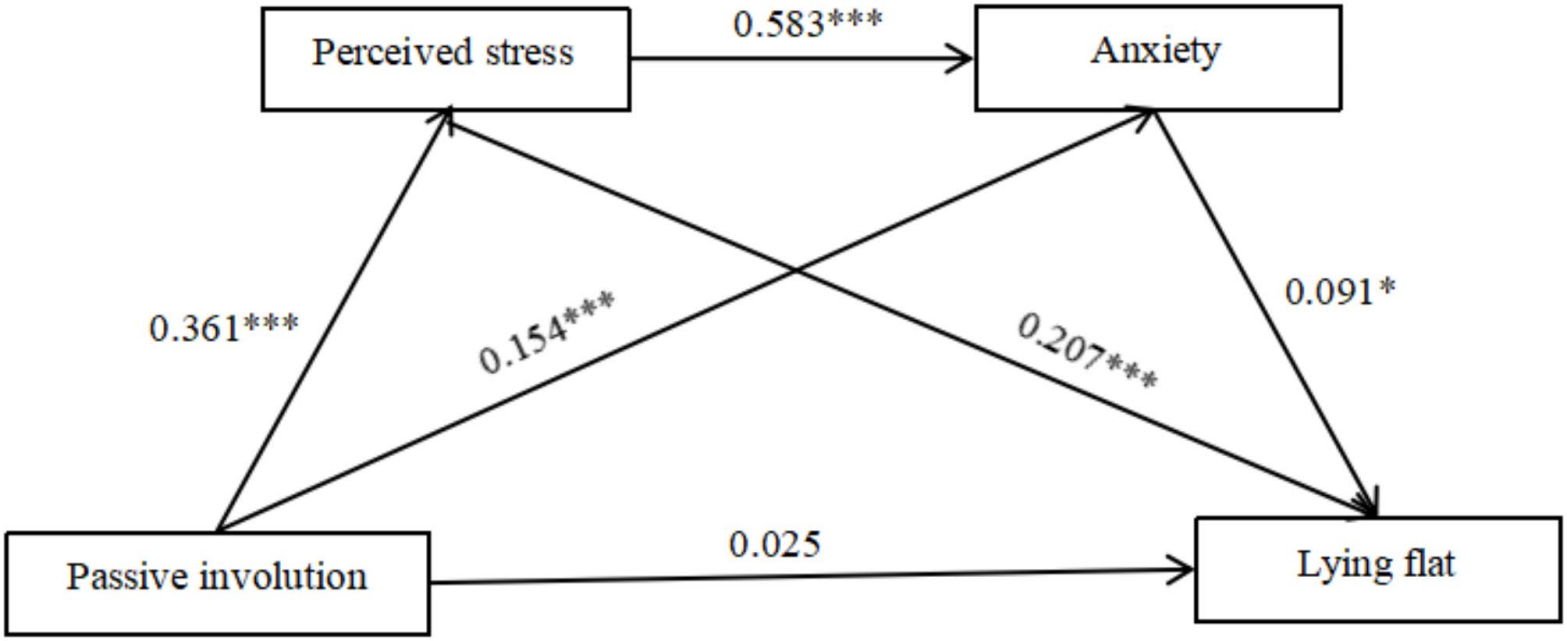
Sequential mediation of perceived stress and anxiety between active involution and lying flat

We adopted Model 6 of the PROCESS macro to test the sequential mediation model of perceived stress and anxiety. As Table 3 illustrates, passive involution positively predicted perceived stress (*β* = 0.361, *P* < 0.001). Passive involution and perceived stress positively predicted anxiety (*β* = 0.154, *P* < 0.001; *β* = 0.583, *P* < 0.001). Perceived stress and anxiety positively predicted lying flat (*β* = 0.207, *P* < 0.001; *β* = 0.091, *P* < 0.05). Passive involution was associated with lying flat (*β* = 0.133, *P* < 0.001). However, when the mediators (perceived stress and anxiety) were entered into the model, the direct effect was not significant (95% CI [-0.024, 0.055]**).** The total indirect effect was 0.108 and significant (95% CI [0.077, 0.140]). Specifically, passive involution significantly independently predicted lying flat via perceived stress (effect size = 0.075, 95% CI [0.042, 0.113]). The indirect effect of passive involution on lying flat through anxiety was also significant (effect size = 0.014, 95% CI [0.000, 0.031]). Passive involution significantly predicted lying flat via perceived stress and anxiety (effect size = 0.019, 95% CI [0.000, 0.039]). Taken together, H2a, H3 and H4 were supported. Figure 2 displays the two-mediator sequential model.

**Table 3:** Regression analysis of variable relationships in sequential mediation model

Outcome variable	Predictor variables	*R*	*R*²	F	β	t
Perceived stress		0.363	0.132	50.547***		
	Age				-0.031	-1.054
	Gender				0.014	0.477
	Passive involution				0.361	12.215***
Anxiety		0.654	0.427	186.045***		
	Age				0.014	0.596
	Gender				-0.026	-1.079
	Passive involution				0.154	5.997***
	Perceived stress				0.583	22.661***
Lying flat		0.311	0.097	21.351***		
	Age				0.077	2.540*
	Gender				-0.110	-3.640**
	Passive involution				0.025	0.767
	Perceived stress				0.207	5.202***
	Anxiety				0.091	2.288*

## Discussion

In recent years, involution and lying flat have become popular in China, and lying flat is often seen as a rebellion against involution. Education is one of the areas with a high incidence of involution. Should involution lead to lying flat in college students? In other words, which students will lie flat after involution and which students will still not lie flat? Therefore, this study focused on college students to explore the direct effect of involution on lying flat and the mediating effect of perceived stress and anxiety. We found that perceived stress and anxiety sequentially mediated the relationship between passive involution and lying flat; perceived stress mediated the relationship between active involution and lying flat.

### The positive influence of passive involution on lying flat

Consistent with H1b, passive involution positively predicted lying flat, which validated the idea that involution leads to lying flat in networks and previous research [22]. It should be noted that the standardized coefficient of passive involution on lying flat (*β* = 0.133) is lower than that reported by Liu and Qin in their study of corporate employees (*β* = 0.259) [48]. This may be because managers are committed to creating various pressures in order to motivate employees to create more value. Compared with college students, corporate employees experience a more profound sense of involution and are also more likely to adopt the “lying flat” behavior [49]. However, after considering perceived stress and anxiety, passive involution does not directly lead to lying flat but rather influences lying flat through perceived stress and anxiety. The total indirect effect was 0.108, with the effect size through perceived stress being 0.075, through anxiety being 0.014, and through the sequential mediation of perceived stress and anxiety being 0.019. These effect sizes indicate that perceived stress and anxiety play important mediating roles between passive involution and lying flat. Although the effect sizes are not large, they reveal the underlying mechanisms of psychological factors in this process. In particular, in populations where passive involution is prevalent, small changes in perceived stress and anxiety may significantly alter the incidence of lying flat behavior.

### Perceived stress mediated the relationship between passive involution and lying flat

Perceived stress mediated the relationship between passive involution and lying flat. First, passive involution can cause students to experience high stress, which is in line with previous study [50]. Limited social resources gradually lead to an implicit social norm of irrational competition [51]. After seeing peers’ involution behaviors (e.g., taking some courses, getting up early, studying in their spare time), passive involutionists are compelled to follow involution for fear of being left behind [3]. They experience upward comparisons. According to social comparison theory, such upward comparisons tend to result in high levels of perceived stress [29]. This process also tends to bring about obstructive stress. Hindrance stress arises from obstacles and constraints that impede progress [28], which is a hallmark of passive involution. This is distinct from challenge stress, which is associated with opportunities for growth and is more relevant to active involution. In the context of passive involution, the pressure to keep up with peers and the fear of falling behind create significant hindrance stress, leading to elevated levels of perceived stress.

Second, passive involution is characterized by avoidance motivation. Faced with the pressure of upwards comparisons, they tend to lose confidence in the future. They turn to lie flat to alleviate stress by giving up excessive effort and lowering their self-expectations [17]. This strategy is a form of psychological defence mechanism aimed at alleviating the overwhelming stress they experience. Previous study also showed that the greater the perceived stress is, the more likely students are to lie flat [52]. Therefore, passive involution may intensify the level of perceived stress, leading to the adoption of lying flat as a coping strategy.

### Anxiety mediated the relationship between passive Involution and lying flat

Anxiety also plays a mediating role between passive involution and lying flat. First, the path from passive involution to anxiety parallels previous research [3]. One possible explanation is that irrational competition may lead to anxiety and reduce mental health [53]. Another explanation is that students who are dominated by external motivation can experience high levels of anxiety [54]. Passive involutionists engage in competition due to the involution atmosphere and peer pressure, which are likely to cause intense anxiety.

Second, high anxiety can induce avoidance behaviors in a variety of areas [37, 39]. For example, students with high exam anxiety often focus on avoiding failure [36]. As quality of life is affected by excessive anxiety and the sense of futility brought about by inefficient competition, college students eventually embrace the attitude of lying flat. In summary, passive involutionists are prone to suffer from excessive anxiety and tend to adopt lying flat behavior to restore well-being.

### Perceived stress and anxiety mediated the relationship between passive involution and lying flat

Passive involution also influences lying flat through the sequential mediating effect of perceived stress and anxiety. A previous study revealed that perceived stress is a strong predictor of anxiety [55]. In this study, the standardized beta of the effect of perceived stress on anxiety (*β* = 0.58) is close to that reported by Ghorbani et al. in their research on 197 American undergraduates (*β* = 0.65) [41]. When individuals perceive stress, they often experience uncontrollable uneasiness and worry that affects their mental health [56]. Zhang found that perceived stress at work tends to negatively affect employees’ emotional experiences, which in turn induces withdrawal behavior [49]. Taken together, passive involutionists are prone to perceive high stress, which subsequently increases lying flat behavior through intense anxiety.

### The negative influence of active involution on lying flat

Active involution not only directly predicts lying flat in a negative direction but also indirectly predicts it via perceived stress. This negative prediction, whether the effect size is large or small, provides empirical evidence that challenges the notion that involution is a primary cause of lying flat. The negative association between active involution and lying flat is largely attributable to the characteristics of active involutionists. These students endorse the social norms of involution and actively adopt competitive behaviors for their own need for achievement and rewards [57]. They are goal-oriented, engage in effort behavior early, and achieve their goals relatively easily. Although they consume a large amount of resources in inefficient competition, they may regard the progress they have made as important new resources that can effectively compensate for the resources they have consumed in their involution and thus continue to maintain their involution behavior. Thus, they have a low willingness to lie flat.

### Perceived stress mediated the relationship between active Involution and lying flat

Active involution also influences lying flat through perceived stress. A previous study revealed that students are better able to adjust to college life when their level of involution matches their involution atmosphere [13]. Active involutionists can balance their interests and learning, compete and enjoy themselves, and have more opportunities to maximize their benefits [15]. Involvement is no longer burdensome or stressful for them. Therefore, college students with high levels of active involution may experience less stress. As mentioned above, there is a positive relationship between perceived stress and lying flat. In summary, college students with greater active involution perceive a lower degree of stress, leading to a lower probability of lying flat.

### Limitations and further research

Several limitations should be considered. First, the sample in this study was limited to college students, which limits our ability to generalize the findings to other stages of education, such as the secondary and postgraduate education stages. Second, although the mediating effect in this study reached statistical significance, it should be noted that its practical impact may be limited. This study is a cross-sectional design and could not establish causality. Longitudinal studies are needed to further explore the causal relationship between active/passive involution and lying flat. Thirdly, the sample size of our study is slightly larger than necessary. With an increased sample size, there is a risk of obtaining smaller effect sizes. Fourth, the lack of a significant correlation between passive involution and anxiety may be attributable to two potential reasons. One possibility is that there is genuinely no association between these two variables. The other possibility is that there may be a suppression effect. Future research could explore whether a variable appears to act as a suppressor in the relationship between active involution and anxiety.

## Conclusion

Despite these limitations, our study constitutes a critical step in examining the mechanism of the relationship between involution and lying flat. Their relationship is not that involution inevitably leads to lying flat, as mentioned in previous studies; rather, it depends on whether different types of involution cause perceived stress and anxiety. College students with passive involution experience greater perceived stress and anxiety, which can increase their lying flat behavior. However, those with active involution experience less perceived stress, which can decrease their lying flat behavior. In conclusion, the key to whether college students adopt “lying flat” behavior in academic learning lies in the passivity of involution and negative feelings.

The importance of this study is to provide empirical support for the relationship between involution and lying flat. Although it needs to be replicated, this study’s findings refute the view that involution inevitably leads to lying flat and have significant practical implications. Firstly, we should not oppose involution, but promote college students to take the initiative to involution. Colleges should pay more attention to students with a high level of passive involution and help them to reduce perceived stress and anxiety, which is likely to reduce the prevalence of lying flat. Secondly, college students should moderately reduce their focus on others and avoid excessive comparisons with peers. Instead, they should choose their college life based on their own talents, interests and strengths, and compare themselves more with their past selves to track their personal growth. Thirdly, when organizing activities and competitions, colleges should establish the guiding principle of “shifting from horse racing to horse selecting.” Based on each student’s performance, they provide feedback and guidance to facilitate their growth.

## Data Availability

The data that support the findings of this study are available from the corresponding author, upon reasonable request.
